# Design and Analysis of a Hollow Metallic Microlattice Active Cooling System for Microsatellites

**DOI:** 10.3390/nano12091485

**Published:** 2022-04-27

**Authors:** Junming Chen, Longquan Liu, Wenjun Xu, Xiaobin Huang, Haoqiang Sheng

**Affiliations:** School of Aeronautics and Astronautics, Shanghai Jiao Tong University, Shanghai 200240, China; cjm1997@sjtu.edu.cn (J.C.); xuwenjun@sjtu.edu.cn (W.X.); xbhuang@sjtu.edu.cn (X.H.); shenghaoqiang@sjtu.edu.cn (H.S.)

**Keywords:** hollow microlattice, heat dissipation, low weight, liquid Ga, microlattice cooling system, microsatellites

## Abstract

Microsatellites have stringent demands for thermal dissipation systems with high efficiency but low weight, which is a difficult combination to obtain using current technologies. The design method of a new cooling system consisting of hollow metallic microlattice material filled with liquid is developed and proposed, and its heat dissipation performance is analyzed through experimental tests and numerical simulations. Through the analysis results of the influences of the microstructures of the hollow microlattice material, it is found that the effective coefficient (the number of channels taking part in convection) has the highest influence on the heat dissipation performance. Numerical simulation results illustrated that the heating surface temperature can be reduced to 301.7 K through special design, which can meet the heat dissipation requirement of most microsatellites. The new microlattice cooling system in this study improves heat dissipation performance while having very low structural weight, thus providing a feasible substitute for thermal control systems in microsatellites.

## 1. Introduction

With the rapid development of space technologies, artificial satellites, space stations, space shuttles, and other spacecrafts have been employed in various tasks for many years. However, their high cost has limited their further application. In this context, microsatellites have been developed rapidly in order to increase their commercial value [1,2]. Compared with large satellites, microsatellites have many advantages, such as small size, light weight, short research cycle, and low cost [3,4]. However, the high electronic integration of microsatellites also brings some limitations to the application of microsatellites. For example, the power-consuming devices inside the microsatellites—such as power controllers, battery arrays, antennas, etc.—greatly deplete the lives of devices if the generated heat in not dissipated in a timely manner [5]. In many monitoring microsatellites, the supplied power generally needs to be controlled within 5 W, and the temperature is required to be less than 323 K [6,7], which is very stringent for most microsatellites. Thus, heat dissipation systems are necessary for microsatellites.

Thermal control systems can be generally divided into active thermal control systems and passive thermal control systems. Compared with the passive method, active thermal control has the characteristics of high adaptability and regulation precision [8]. A pumped fluid loop is one of the active thermal control methods whose working principle is to take away the excess heat by the flow of liquid with the help of radiators and pumps [9]. Similarly to the principle of a pumped fluid loop, microchannel heat sinks enhance heat transfer by introducing a microchannel structure to achieve higher cooling efficiency, which is usually used in spacecraft with high power consumption such as phased array antennas [10,11]. However, the additional package weight of microchannel heat sinks is non-negligible, making it difficult to apply them in microsatellites which have very stringent requirements on weight and volume [12,13]. Therefore, it is essential to develop a new type of lightweight thermal control system for microsatellites.

Hollow microlattice material (also called ‘nanolattice material’) is an ultralight multi-functional nanomaterial, which is assembled by thin-walled tubes according to different configurations [14,15,16,17]. It will be a new generation of lightweight active cooling system by filling the hollow tubes with fluid and using the peristaltic pump to drive the fluid to flow. Since liquid metal gallium (Ga) possesses high thermal conductivity and good fluidity, it has been studied and used in many heat dissipation fields [18,19,20]. The fluidity of gallium means that it cannot be used as a heat dissipation material alone, so it is usually used as an interface material or in combination with structural materials in existing studies. Ji et al. [21] prepared a sandwich structure by filling a novel gallium-based liquid metal as an interfacial material in the middle of an aluminum substrate. The results illustrated that the new liquid metal can not only avoid its reaction with the aluminum substrate, but also greatly reduce the contact thermal resistance, which was an ideal material for the thermal conductive interface. Xu et al. [22] prepared a heat sink by combining liquid metal gallium and foam metal together. The results showed that gallium was much more efficient compared to paraffin and it achieved weight reduction with the same target of heat dissipation.

In this study, a hollow microlattice material embedded with Ga is developed and investigated as a cooling system for the first time. Theoretical analyses, experimental tests and numerical simulations are conducted to analyze the heat dissipation performance of the new cooling system. The designability of the hollow microlattice ensures that it can achieve the functionality of heat dissipation while meeting the requirements of weight and volume [15], providing a new concept for designing lightweight active cooling system.

## 2. Design and Theoretical Analysis

**Figure 1:**
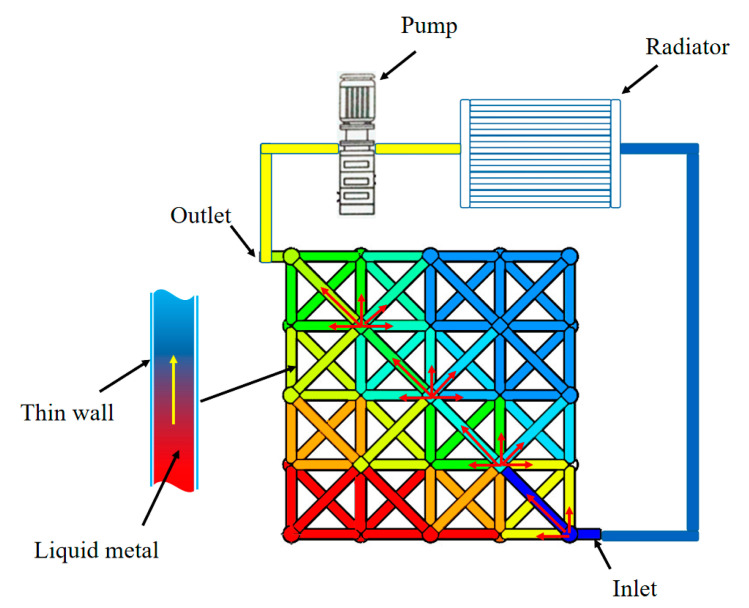
The design concept of the microlattice cooling system.

The concept of the proposed hollow microlattice cooling system is illustrated in Figure 1. The hollow microlattice is an assembly of thin metallic tubes that can be used as a ‘container’ to be filled with liquid. Liquid metal can flow inside the interconnected tubes and transfer heat without adding too much structural weight. A peristaltic pump can be connected to the hollow microlattice to drive the liquid metal to flow and take away the heat quickly and thus further enhance heat dissipation performance.

In order to simplify the theoretical analysis of the model, the following basic assumptions are made: (1) the influence of thermal radiation is disregarded; (2) the liquids are incompatible, laminar, and in a steady state; and (3) kinematic viscosity, density, thermal conductivity, and Prandtl number are constant and do not vary with temperature.

**Figure 2:**
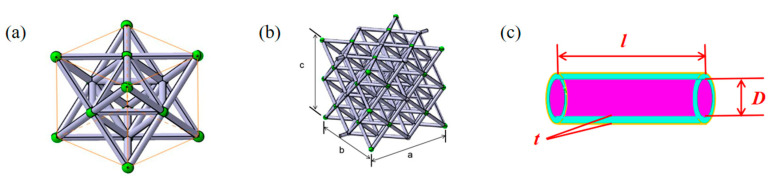
The (**a**) unit-cell, (**b**) structure of the microlattice material, and (**c**) hollow tube.

A single octet cell (Figure 2) is selected for analysis consisting of 24 hollow tubes.

From Figure 2, it can be seen that the unit cell is composed of 24 thin tubes with a diameter of *D*, a thickness of *t*, and a length of *l*. Thus, the mass of one unit-cell of hollow microlattice filled with liquid can be calculated as

(1) $$m=24\times \rho_{w}\times \pi \left(Dt+t^{2}\right)\times l+6\times \rho_{i}\times \pi D^{2}l$$

where *ρ_w_* and *ρ_i_* are the densities of the thin wall and fluid inside; and *D*, *l*, and *t* are the diameter, length, and thickness of the hollow tube respectively.

The heat transferred per degree Celsius by convection is determined by the convective heat transfer coefficient and the intersectional area of the flow channel, which is defined as *hA*. The inner fluid is in laminar flow state and forced convective heat transfer, and the convective heat transfer coefficient can be calculated as

(2) $$h_{i}=\frac{Nuk}{D}$$

where *Nu* represents the intensity of convective heat transfer and can be calculated by Sieder–Tate equation as

(3) Νu=1.86(RePrlD)13(ηfηw)0.14

where Re represents the flow condition of the fluid and it can be calculated as

(4) Re=uDν

Thus, heat transferred rate by convection *hA* can be expressed as

(5) $$hA=p\times u^{\frac{1}{3}}D^{\frac{2}{3}}l^{\frac{2}{3}}\times 1.86\pi {\left(\frac{\mathrm{Pr}}{\nu }\right)}^{\frac{1}{3}}{\left(\frac{\mu_{f}}{\mu_{w}}\right)}^{0.14}k$$

wherein, these parameters, Pr, *v*, *μ*, and *k* are constants dependent on the nature of the fluid, which are shown in Table 1. *p*, *u*, *D* are variables, where *p* is the effective coefficient, which represents the number of the tubes contributed to convective heat transfer; and *u* is the flow rate of the fluid inside tube. With the parameters determined in Table 1, the influences of the three variables on *hA* are studied. The initial values of three variables are defined as *D* = 1 mm, *u* = 5 cm/s, *p* = 1.

**Figure 3:**
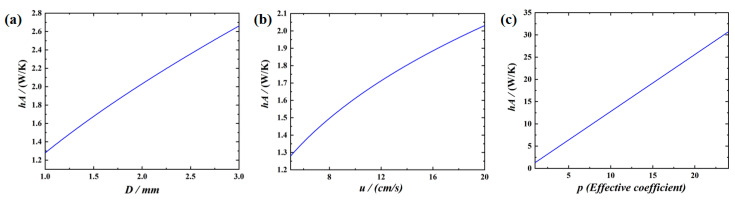
Effects of (**a**) diameter; (**b**) fluid rate of the liquid; (**c**) effective coefficient on *hA*.

Through theoretical analysis, the influences of the parameters on the cooling effect are shown in Figure 3 by calculating Equation (5). Figure 3a illustrates the influence of the diameter of the tube on heat transfer. From Figure 3a, it can be seen the heat transfer is increased with the increase in the diameter of the tubes. This is because the increased diameter results in a larger value of *Nu* and then enhances the convective capacity of the internal fluid greatly.

The effect of the flow rate of the liquid metal is shown in Figure 3b. It can be seen from the Figure 3b that the *hA* is increased with the increase in the flow rate of internal fluid, which is due to the increased flow rate resulting in the rise of convective heat transfer coefficient, and thus the heat transfer performance of the system is increased. It can be clearly seen from Figure 3c that the value of *hA* increases significantly with the increase in the effective coefficient.

From the comparisons of the Figure 3a–c, it can also be seen that the effective coefficient has higher influence than the diameter of the tubes and the flow rate of fluid. Therefore, in the design of the cooling system, emphasis should be placed on the design of the number of fluid channels to allow more tubes to take part in convective heat transfer.

## 3. Numerical Simulation and Validation

### 3.1. Heat Dissipation Model and Grid Independence

**Figure 4:**
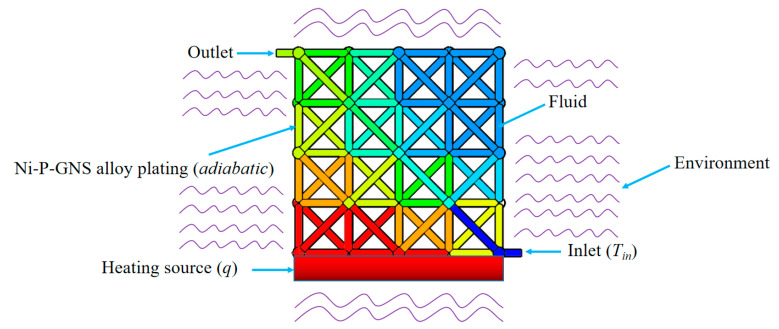
Simulation model sketch.

Simulation model of the heat dissipation performance of the cooling system was established using the finite element code, Fluent. The simulation model was shown in Figure 4, where the cooling system included 8 Octet cells and the dimensions were 28 × 28 × 28 mm. The microlattice cooling system was supposed to be heating at a constant heat flux density of 2 W/cm^2^. The tubes of the hollow lattice were filled with liquid metal, where the velocity of the liquid metal was variable with the temperature of 293 K. The Ni-P-GNS thin film was treated as s wall and its thickness was set as 10 μm, and the thermal mode was selected as convection. The material parameters of liquid Ga and solid Ni-P-GNS thin film were shown in Table 2.

**Figure 5:**
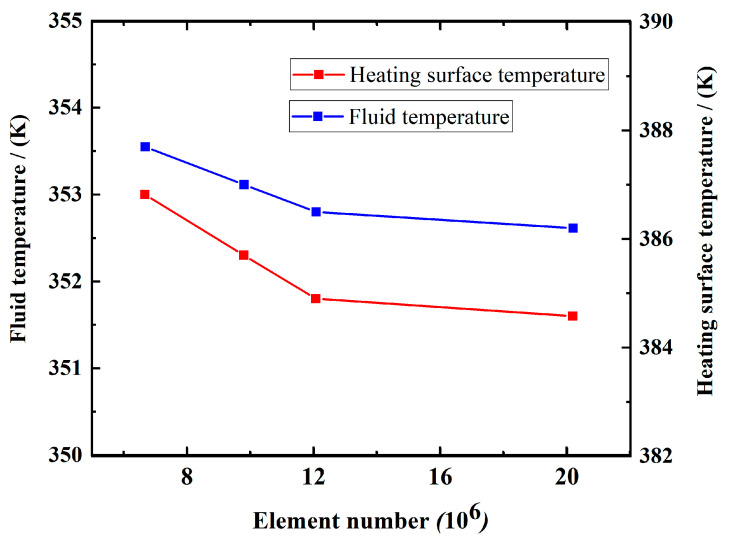
Average temperature of heating surface and fluid for various element numbers.

Figure 5 illustrated the analysis of the average temperature of the heating surface and fluid for different element numbers. The average temperature gradually converges as the element number is more than 12,073,592, thus demonstrating the grid independence.

The heat dissipation performance of the microlattice cooling system was characterized by comparing the average temperature of heating wall and calculating the average convective heat transfer coefficient of the fluid [23]. In this condition, the Ni-P-GNS wall was set as adiabatic condition to simulate the environment of outer space. The heat carried away by the liquid metal *q*_1_ can be expressed by

(6) $$q_{1}=h_{f}A_{c}\Delta T_{1}=h_{f}A_{c}(T_{heating}-T_{f})$$

Additionally, *q*_1_ can be calculated by the equation

(7) $$q_{1}=c_{f}m_{f}\Delta T=c_{f}\rho_{f}A_{inlet}v_{inlet}(T_{out}-T_{in})$$

Therefore, the average convective heat transfer coefficient can be calculated by

(8) $$h_{f}=\frac{c_{f}\rho_{f}A_{inlet}v_{inlet}(T_{out}-T_{in})}{A_{c}(T_{heating}-T_{f})}$$

where *A_c_* = 62.48 cm^2^ (the effective area of the cooling system), *A_inlet_* = 0.0075 cm^2^ (the area of the inlet), *v_inlet_* = 5–15 cm/s (the velocity of the inlet), *T_in_* = 293 K. *T_heating_*, *T_f_*, and *T_out_* represented the average temperature of the heating surface, fluid, and outlet of the fluid respectively.

### 3.2. Model Validation

#### 3.2.1. Fabrication of the Microlattice Cooling System

**Figure 6:**
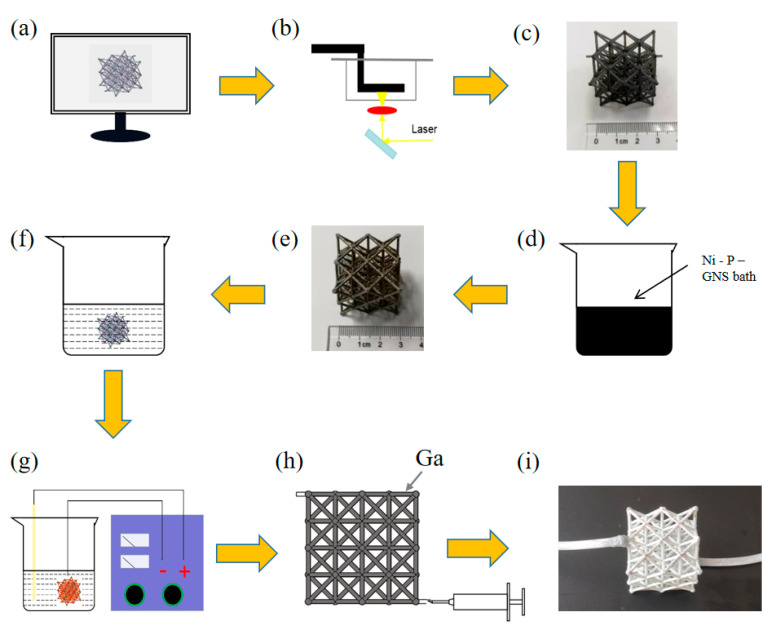
Fabrication of the microlattice cooling system. (**a**) Computer micro-architectural design. (**b**) 3D printing. (**c**) Polymer template. (**d**) Chemical plating. (**e**) Ni-P-GNS thin film template. (**f**) Chemical etching of template removal. (**g**) Electro-coppering. (**h**) Liquid Ga injection. (**i**) Microlattice cooling system.

The fabrication process of the cooling system is shown in Figure 6. Firstly, a stereolithography-based 3D printing method (Figure 6b) was employed to fabricate the polymer template using Somos Taurus (Royal DSM) photosensitive resin. Secondly, the polymer template was cleaned by ultrasonication to remove possible contamination on the surfaces. Then the template was activated with a colloidal palladium solution in 10 min at 40 °C and put into 50 g/L NaOH solution for 10 s at room temperature to expose palladium nanoparticles as catalyst. Thirdly, the Ni-P electroless plating solution (Guangzhou Yishun Chemical Co., Ltd., Guangzhou, China) was blended with the prepared dispersed graphene nanosheet (GNS) solution and stirred until a uniform Ni-P-GNS composite electroless plating was gained. Then the template was immerged in 90 °C water for 3 min and put into the composite Ni-P-GNS solution in 90 °C for 90 min. After plating, some outer nodes of samples were sanded to expose the resin substrate, the internal resin was dissolved by the etching solution which comprise 20 g/L NaOH and 700 mL/L ethanol bathed in 60 °C water for 24 h to remove the template. Finally, the melting phase change materials gallium (Ga) were injected into the hollow microlattice and a peristaltic pump was connected, a whole microlattice cooling system was prepared with the structural weight of 0.45 g.

#### 3.2.2. Test Setup

**Figure 7:**
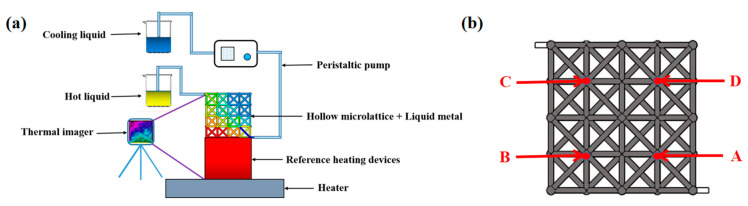
(**a**) Test setup and (**b**) test point of the heat dissipation performance.

The test setup of microlattice cooling system is shown in Figure 7a. The copper block was being heated by a heater, which was treated as a heating device. The test sample was placed on the heated copper block and was connected with a peristaltic pump. An infrared thermal imager (Fotric 348X) was used to measure and record the temperature changing process of the copper block and the microlattice material during heating. The average surface temperature of the copper block *T_a_* was measured to characterize the heat dissipation performance of the microlattice cooling system.

#### 3.2.3. Comparison between Test and Simulation Results

The temperature of different locations (Point A, B, C, and D shown in Figure 7b) of the microlattice cooling system in the experiment was selected for comparison with the simulation results, which was shown in Table 3. In this case, the heating source was set to be a temperature of 337 K, which is the steady temperature of the reference heating source in the experiment. Additionally, the Ni-P-GNS wall was set as convection with heat transfer coefficient 10 W/(m^2^·K) under the temperature of 293 K to simulate the environment of air in the experiment and the inlet velocity was set as 5 cm/s. It was obvious that the simulation data were quite consistent with theoretical results and the maximum error was 1.53%, therefore the numerical model was credible.

## 4. Results and Discussion

### 4.1. Experimental Results and Discussion

**Figure 8:**
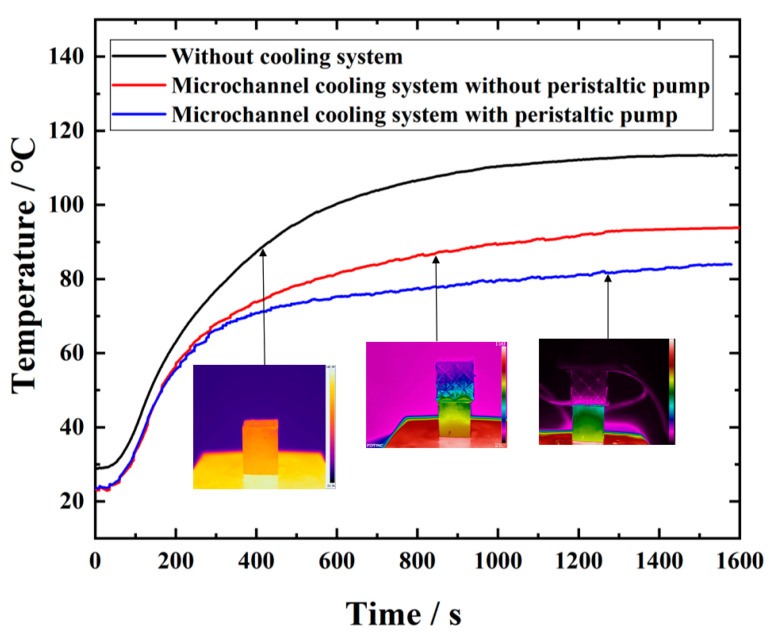
Test results of the heat dissipation performance of the cooling system.

Figure 8 shows the average temperature change of copper block during the heating process, and Table 4 exhibits the final stable temperature of the copper block. It is evident from Figure 8 and Table 4 that there is a significant difference in the heat dissipation performance of the different cooling methods. Compared with the case without a cooling system, the surface temperature of the copper block is decreased by 29.5 K using the microlattice cooling system from this experiment.

In the absence of a cooling system, the heat of the reference heating devices can only be dissipated into the air via weak convection, for which the efficiency is too limited. With the presence of the new microlattice cooling system, the heat dissipation mainly relies upon the high thermal conductivity and natural convection of liquid Ga without the driving effect of the peristaltic pump, whose cooling efficiency is average. However, with the driving effect of the peristaltic pump, the convective heat transfer coefficient is large due to the contribution of microchannels, and its heat dissipation efficiency is improved substantially compared to the previous one.

### 4.2. Numerical Simulation Discussion

Table 5 shows the numerical simulation results at different working conditions. It is clear from Table 5 that with the diameter and inlet velocity increasing, the convective heat transfer coefficient of the fluid is significantly enhanced and the temperature of the heating wall is reduced, indicating a better cooling effect. It is worth mentioning that the values of the heating surface temperature and the convective heat transfer coefficient show that the diameter and the inlet velocity has an approximate influence on the heat dissipation performance, which is consistent with the theoretically derived results in Figure 3a,b.

**Figure 9:**
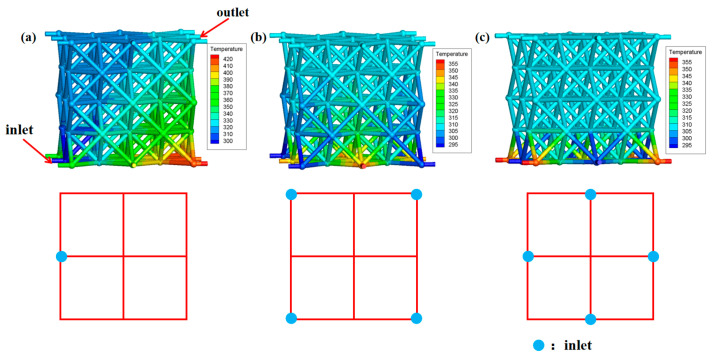
Influence of liquid inlet locations (**a**) one inlet in the middle; (**b**) four inlets at the corner; and (**c**) four inlets in the middle on heat dissipation performance.

Figure 9 illustrates the influence of the fluid inlet location on heat dissipation performance of microlattice cooling system (*v_inlet_* = 5 cm/s, *D* = 1 mm). As shown in Figure 9a, the inhomogeneity of temperature distribution on the heating wall would have a negative effect on the heating element itself, so it is necessary to design a reasonable inlet/outlet channel, which is consistent with the analysis of the effective coefficient in the theoretical derivation.

Table 6 exhibits the results of the heating surface temperature and convective heat transfer coefficient under different fluid inlet locations. According to the simulation results, the temperature of heating surface is significantly reduced by reasonable design of inlet location, which could be attributed to the increase in the effective coefficient (theoretical derivation). Additionally, the temperature of the heating surface can be further reduced to 301.7 K (*v_inlet_* = 10 cm/s, *D* = 1.2 mm), which can greatly meet the heat dissipation needs of the heating sources in most microsatellites [24]. Through the reasonable arrangement of the inlet location, more fluid in tubes will contribute to convection, thus enhancing the heat transfer capacity of the fluid.

## 5. Conclusions

(1) The design methods and analysis techniques for heat dissipation performance of hollow microlattice active cooling systems filled with liquid metal were developed considering the microstructural features of the hollow microllatice material.
(2) The heat dissipation performance of the new microlattice cooling system is mainly related to the diameter of the tube, the flow rate of the fluid, and the location of the fluid inlets. Among these factors, the inlets’ locations have the greatest influence.
(3) When the structural parameters of microlattice are set as *D* = 1.2 mm, *l* = 10 mm, the flow parameters endow four inlets with *v_inlet_* = 10 cm/s, the convective heat transfer coefficient can reach up to 293.9 W/(m^2^·K) and the temperature of the heating surface can be reduced to 301.7 K under a heat source to a power of 6 W, with the condition that the structural weight is only 0.45 g.
(4) The newly developed cooling system is suitable for the temperature control of most monitoring microsatellites.

## Figures and Tables

**Table 1:** Values of the constant.

Parameters	Pr	*v*/m^2^·s^−^^1^	*k*/W/(m·K)	*μ*/kg/(m·s)	*l*/mm
	0.024	3.1 × 10^−7^	29.4	0.00183	10

**Table 2:** Parameters of the materials.

	Density (kg/m^3^)	Specific Heat (J/(kg·K))	Thermal Conductivity (W/(m·K))	Viscosity (kg/(m·s))
Ga	5910	410	29.4	0.00183
Ni-P-GNS	8800	493	55.8	

**Table 3:** Comparison of test and numerical results (K).

Location	A	B	C	D
Experiment	326	330	309	317
Simulation	331	332	313	318
Error (%)	1.53	0.61	1.29	0.32

**Table 4:** Results of the experiment.

	Without Cooling System	Microlattice Cooling System without Peristaltic Pump	Microlattice Cooling System with Peristaltic Pump
*T_a_*/K	386.5	366.8	357.0

**Table 5:** Simulation results under different inlet velocities and diameters.

	*T_f_* (K)	*T_out_* (K)	*q*_1_ (W)	*T_heating_* (K)	*h_f_* (W/(m^2^·K))
*v_inlet_* = 5 cm/s, *D* = 1 mm	351.8	351.1	5.3	386.5	24.2
*v_inlet_* = 5 cm/s, *D* = 1.2 mm	333.6	334.2	5.5	357.2	32.1
*v_inlet_* = 10 cm/s, *D* = 1 mm	324.9	325.5	5.9	352.7	33.8
*v_inlet_* = 15 cm/s, *D* = 1 mm	315.0	314.6	5.9	338.2	40.6

**Table 6:** Simulation results under different fluid inlet locations.

Locations	*T_heating_* (K)	*h_f_* (W/(m^2^·K))
One inlet in the middle	372.3	24.2
Four inlets at the corner	325.9	67.1
Four inlets in the middle	320.9	91.3

## Nomenclature

*A*	area (mm^2^)
*D*	diameter of one tube of microlattice (mm)
*h*	convective heat transfer coefficient (W/(m^2^·K))
*k*	thermal conductivity (W/(m·K))
*l*	length of the tube of microlattice (mm)
Nu	Nusselt number
*p*	effective coefficient
Pr	Prandtl number
*q*	heat flux density (W/cm^2^)
Re	Reynolds number
*T*	temperature (K)
Δ*T*	temperature difference (K)
*t*	thickness of hollow tube (μm)
*u*	flow rate of fluid (cm/s)
Greek symbols	
*ν*	kinematic viscosity(m^2^/s)
*μ*	dynamic viscosity (kg/(m·s))
*ρ*	density (kg/m^3^)
Subscript	
*f*	fluid
*in*	inlet
*out*	outlet
*w*	wall
